# Environment and Lifestyle: Their Influence on the Risk of RA

**DOI:** 10.3390/jcm9103109

**Published:** 2020-09-26

**Authors:** Carine Salliot, Yann Nguyen, Marie-Christine Boutron-Ruault, Raphaèle Seror

**Affiliations:** 1 Centre for Research in Epidemiology and Population Health, (CESP), INSERM U1018, Université Paris-Sud, F-94800 Villejuif, France; carine.salliot@chr-orleans.fr (C.S.); yann.nguyen2@aphp.fr (Y.N.); marie-christine.boutron-ruault@gustaveroussy.fr (M.-C.B.-R.); 2 Rheumatology Department, Centre Hospitalier Régional d’Orléans, 45100 Orléans, France; 3 Centre of Immunology of Viral Infections and Auto-immune Diseases (IMVA), INSERM U1184, Université Paris-Sud, F-94270 Le Kremlin Bicêtre, France; 4 Department of Internal Medicine, AP-HP. Nord, Hôpital Beaujon, Université de Paris, F-92100 Clichy, France; 5 Rheumatology Department, AP-HP, Hôpitaux universitaires Paris-Saclay—Hôpital Bicêtre, F-94270 Le Kremlin Bicêtre, France

**Keywords:** rheumatoid arthritis, environmental factors, risk, gene–environment interaction, rheumatoid arthritis subsets, smoking, inhalant exposure, hormonal exposure, diet

## Abstract

Background: Rheumatoid arthritis (RA) is a complex disease in which environmental agents are thought to interact with genetic factors that lead to triggering of autoimmunity. Methods: We reviewed environmental, hormonal, and dietary factors that have been suggested to be associated with the risk of RA. Results: Smoking is the most robust factor associated with the risk of RA, with a clear gene–environment interaction. Among other inhalants, silica may increase the risk of RA in men. There is less evidence for pesticides, pollution, and other occupational inhalants. Regarding female hormonal exposures, there is some epidemiological evidence, although not consistent in the literature, to suggest a link between hormonal factors and the risk of RA. Regarding dietary factors, available evidence is conflicting. A high consumption of coffee seems to be associated with an increased risk of RA, whereas a moderate consumption of alcohol is inversely associated with the risk of RA, and there is less evidence regarding other food groups. Dietary pattern analyses (Mediterranean diet, the inflammatory potential of the diet, or diet quality) suggested a potential benefit of dietary modifications for individuals at high risk of RA. Conclusion: To date, smoking and silica exposure have been reproducibly demonstrated to trigger the emergence of RA. However, many other environmental factors have been studied, mostly with a case-control design. Results were conflicting and studies rarely considered potential gene–environment interactions. There is a need for large scale prospective studies and studies in predisposed individuals to better understand and prevent the disease and its course.

## 1. Introduction

The immune onset of rheumatoid arthritis (RA), so called “preclinical phase” of the disease, might occur several years before the first symptoms of RA, with the development of autoimmunity as evidenced by detectable anti-citrullinated peptide antibodies (ACPA) and rheumatoid factors (RF). The “mucosal paradigm” hypothesizes that environmental factors may lead to inflammation of the pulmonary or the gut mucosa, and to locally prime autoimmunity in individuals with genetic predisposal, leading to autoantibody production years before RA onset [1].

The involvement of environmental, dietary, reproductive, and lifestyle factors in the pathogenesis of RA is supported by numerous observations which include the following: two thirds of individuals who develop RA are women, suggesting the role of female hormones; also the latitude gradient influences the incidence of RA and age at onset, and the socioeconomic status and educational levels are consistently associated with the risk of RA [2,3,4,5].

The aim of this study is to review the literature evidence on external and internal exposures (so called “exposome”) associated with the risk of RA and its phenotype, and their interaction with genetic risk factors. First, we discuss cigarette smoking, which is the main environmental risk factor for RA, then, we discuss other inhalants, female hormonal and reproductive factors, and dietary factors.

## 2. Cigarette Smoking

### 2.1. Active Cigarette Smoking

Smoking is the most robust and well documented environmental risk factor associated with RA. A meta-analysis by Di Giuseppe et al. included three prospective cohorts and seven case-control studies [6,7,8,9,10,11,12,13,14,15,16]. A comparison with never smokers showed that those who had 1 to 10 pack-years of smoking had a 26% increased risk of RA (relative risk (RR) = 1.26, 95% CI 1.14–1.39), whereas risk doubled among those with more than 20 pack-years (RR for 21–30 pack years = 1.94, 95% CI 1.65–2.27, and RR for >40 pack-years = 2.07, 95% CI 1.15–3.73). In addition, the risk associated with the highest versus the lowest category of pack-years of smoking was higher for RF-positive RA (RR = 2.47 and 95% CI 2.02–3.02) than for RF-negative RA (RR = 1.58, 95% CI 1.15–2.18).

The risk of RA associated with smoking was generally higher among men than among women [17]. Interestingly, the association between smoking and RA decreased after smoking cessation. Twenty years after smoking cessation, there was no longer any association between smoking and ACPA-negative RA, whereas the association with ACPA-positive RA risk persisted and remained linked to the cumulative dose of cigarette smoking [17,18,19].

### 2.2. Interaction between Smoking and Genetic Risk Factors for Rheumatoid Arthritis (RA)

**Figure 1:**
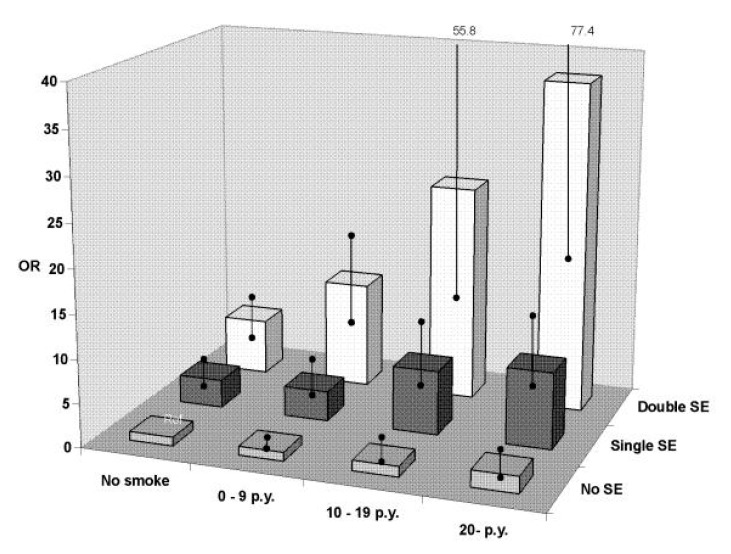
Odds ratios (OR) for ACPA-positive rheumatoid arthritis (RA) and different amounts of smoking (pack-years) in combination with none (no shared epitope (SE)), one (single SE), or two (double SE) copies of SE alleles. The reference group being no smokers without SE alleles. Figures from Källberg et al. Smoking is a major preventable risk factor for rheumatoid arthritis. Estimations of risks after various exposures to cigarette smoke. (Ann. Rheum. Dis. 2011; 70:508-11 [19].)

Numerous studies have investigated the interaction between a well-known genetic risk factor (HLA-DRB1 shared epitope (SE)) and smoking on RA development for both antibody positive and antibody negative RA [19,20,21,22,23,24,25]. Smokers carrying two copies of the SE were at a 21-fold increased risk of ACPA-positive RA as compared with non-smokers carrying no SE copy [25]. Moreover, SE- and smoking-related risk of ACPA-positive RA increased with the intensity of smoking and the number of SE alleles (Figure 1) [19]. These results suggest a strong gene–environment interaction with a dose-response effect for both genetic and environmental factors for the risk of ACPA-positive RA.

To date, the main pathogenetic hypothesis for this interaction concerns the presence of citrulline-modified proteins in the lungs of smokers (due to local mucosal inflammation) leading to a systemic immune response to these citrullinated proteins by ACPA production, preferentially induced in individuals carrying SE genes (having higher affinity for citrullinated peptides) [25,26,27,28].

Multiple other genetic factors have also been associated with RA such as PTPN22, PADI4, CTLA-4, STAT4, etc. [29]. For PTPN22, the risk was stronger for RF positive than for RF negative RA [30,31]. PTPN22 and PADI-4 polymorphisms may also lead to hypercitrullination and may be implicated in ACPA production and the development of RA. Nevertheless, interactions between those genetic risk factors and smoking, and according to RA subsets, remain unclear [32,33,34,35,36].

### 2.3. Passive Smoking, Including Fetal Exposure through Maternal Smoking

Few studies have examined risks associated with passive smoking. In adulthood, passive smoking exposure at work or at home was not associated with RA in three case-control and cohort studies [7,37,38]. In a cohort of French women from the education system, ever-smokers with passive smoking exposure during childhood had a higher risk of RA than smokers with no passive smoking during childhood [39]. In another study, high maternal smoking during pregnancy (>10 cigarettes per day) increased the risk of RA and other inflammatory polyarthritis during childhood only in girls as compared with no maternal smoking (OR = 2.57, 95% CI 1.13–5.89) [40].

However, measurement of passive smoking is challenging and heterogeneous which might explain these discrepancies. Assessment of the effect of passive smoking on RA should also consider active smoking, timing, duration, and intensity of passive exposure.

## 3. Inhaled Exposures Other Than Smoking

Although tobacco consumption has decreased over the last decades, RA incidence remains stable in the USA [41]. Other inhalants may play a role since many non-smokers develop RA, by inducing pulmonary mucosal inflammation and systemic immune response with ACPA production [28]. This chapter summarizes available evidence regarding inhaled occupational exposures (silica, pesticides, and others), and air pollution (Table 1).

### 3.1. Silica

Silica exposure is the second most documented environmental factor associated with the risk of RA. After adjustment for smoking, several cohort and case-control studies reported associations between RA in men and specific occupations such as granite workers, rock drilling, and stone crushing [42,45,48,49,50]. There was only one case-control study that reported an inverse association between silica exposure and RA risk among pottery, sandstone, and refractory material (aluminosilicate or silica) workers [43].

Similar to smoking, silica exposure has been mainly associated with seropositive RA [46,47]. In a Swedish register of silica-exposed male workers in iron foundries, individuals employed for at least one year had an increased risk of seropositive RA as compared with the general population (SIR=1.70, 95% CI 1.01–2.69) increasing to 2.59 (95% CI 0.24–4.76) among highly exposed individuals, with a dose-response relationship [71].

Interestingly, a high risk of ACPA-positive RA was observed among silica-exposed current smokers (OR = 7.36, 95% CI 3.31–16.38), suggesting an interaction between these exposures [46]. Few other studies supported such silica–smoking interaction [48,72]. These results must be taken with caution because smoking duration or intensity was not taken into account.

Thus, there is some evidence that occupational silica exposure in men could be a risk factor for seropositive RA with a dose-response relationship, and that smoking would increase the risk associated with silica exposure. This suggests that silica could share the same pulmonary mucosal inflammation pathway as smoking.

### 3.2. Pesticides

Two large Swedish studies reported no association between occupational pesticide exposures and risk of RA [44,62]. However, in a cross sectional study conducted among male pesticide sprayers in Greece, high pesticide exposure (total number of pesticide applications throughout the lifespan) was associated with RA (all pesticides OR = 43, 95% CI 3.09–600.67; for insecticide OR = 15.29, 95% CI 1.24–189.02; and fungicides OR = 14.3, 95% CI 1.38–150.37) as compared with low exposure [73].

The Agricultural Health Study (Table 1) provided information on ever-use of 50 pesticides with duration and frequency among farmers and spouses. Among male pesticide sprayers, fonofos, carbaryl, and chlorimuron ethyl were associated with increased RA risk, but not DDT or glyphosate. Dose-response associations with RA were observed for atrazine, toxaphene, and fonofos [54]. Among farmers’ spouses, the use of any specific pesticide among the 15 examined pesticides including glyphosate was slightly associated with RA as compared with no exposure (Table 1) [55,56].

In the Women’s Health Initiative Observational Study, residential or workplace insecticide use was associated with RA in post-menopausal women (*p* = 0.0026) [74]. Early-life pesticide exposure and farm residence during childhood increased the risk of adulthood onset of RA in women (Table 1) [57].

These conflicting results, regarding the effect of specific pesticide, may be explained by methodologies of the studies, exposure misclassification (mainly self-reported exposure), timing and frequency of pesticide use, and unmeasured confounding.

### 3.3. Others Inhalant-Related Occupations

Table 1 summarizes other inhaled occupational exposures potentially associated with RA risk. Some occupations would be associated with the risk of RA in men, such as construction workers (with studied exposures to non-silica dust, organic solvents, asbestos, vermiculite, or asphalt) and farmers (with studied exposures to chemical fertilizers, non-gasoline solvent, cleaning solvent, or farm animals) [44,48,56,58,59,60,62,63,64,75].

In men, coal dust has been associated with an elevated risk of RA and 33% of RA has been be attributable to coal mining work [50].

With less evidence, transportation workers (exposed to mineral oil), steel workers, plastic industry workers (exposed to styrene), military workers (exposed to smoke from open-air burn pits), and electronic workers (exposed to potential noxious airborne agents) may have an increased risk of RA (Table 1) [44,52,53,59,61,62,75].

In women, exposure to textile dust has been reported associated with an increased risk of RA with a potential interaction with SE [51].

### 3.4. Air Pollution

Air pollution is a mixture of gas pollutants including ozone (O_3_), carbone monoxide (CO), fine particulate matter (PM_10_, PM_2.5_), nitrogen dioxide (NO_2_), and sulphur dioxide (SO_2_).

Living close to a highway or a major road has been reported to be associated with a 30% increased risk of RA, suggesting a possible association with air pollution [65,66]. Intense dust and smoke exposures after the World Trade Centre’s terrorist attack have been associated with doubling the risk of systemic autoimmune diseases, mostly RA [76]. These results suggest a possible association between RA and air pollution.

However, high levels of exposure to NO_2_, PM_2.5_, PM_10_, and SO_2_ have not been associated with RA (Table 1), except for one study with no adjustment for smoking [66,68,69,70]. O_3_ and CO levels could be associated with RA [66,67].

However, results are still unclear, possibly due to discrepancies in measuring air pollution exposure (residential addresses prior or at diagnosis or traffic and home heating), and methodological limits regarding timing and accounting for confounders (such as smoking and the socioeconomic status).

## 4. Reproductive Factors in Women

The implication of female hormones in the pathogenesis of RA has been supported by numerous observations which include the following: a 2:4 female/male ratio before the age of 50 but below 2:4 after the age of 60, an increased incidence during postpartum, a peak of RA incidence around the age of menopause, and about 50% of RA starts during a woman’s reproductive life. During a woman’s life, some events such as pregnancy, postpartum, breastfeeding, menopause, and the use of exogenous hormones induce changes in female hormonal exposures. Indeed, estrogens and progestogens may have pro-inflammatory or anti-inflammatory effects depending on serum levels and reproductive stage (reproductive life, menopausal transition, and post-menopause). Then, during the menopausal period, the decline of estrogen and progestogen levels is associated with an increase of pro-inflammatory cytokines, such as IL-6, TNFα, and IL-1α [2]. Table 2 summarizes results from cohort and case-control studies, regarding reproductive factors and hormonal treatments.

### 4.1. Ages at Menarche and Menopause

The role of early menarche on the risk of RA is unclear, with two cohorts and three case-control studies providing conflicting results [86]. Early age at menopause (≤44 or 45 years) was associated with increased risk of seronegative RA in two cohort studies and one case-control study [78,79,89]. Two other case-control studies did not find any association between age at menopause and RA risk [14,86].

Recently, Alpizar-Rodriguez et al. reported an increased incidence of ACPA-positive RA with menopause, especially in the early post-menopausal period (i.e., within <6 years after menopause) among women at risk of RA (first-degree relatives of patients with RA). This suggests that the acute decline in ovarian function could contribute to the development of autoimmunity and potentially to an increased risk of RA in women [93].

### 4.2. Parity and Postpartum

During the 12 or 24 month postpartum period, incident cases of RA are more frequent than later on [83,84,94]. The risk seems to be maximal during the first three months after delivery and reduces during the subsequent nine months [82]. This supports a role for hormonal changes during pregnancy or after delivery in RA onset. Because of the short time, it could be questioned whether RA arises de novo or rather RA symptoms arise in women with already triggered autoimmunity.

A high number of pregnancies could reduce the risk of RA [14,80,81,84,86] or could have no impact on the risk of RA [77,78,79,85] (Table 2). In line with those studies, a meta-analysis that included 12 studies demonstrated a borderline significant inverse association between parity (versus nulliparity) and RA (RR 0.90, 95% CI 0.79–1.02), and a significant nonlinear inverse relation between parity number and the risk of RA [95].

Contrarily, a case-control study showed that parous women (versus nulliparous) had an increased risk of seronegative RA in the age group 18–44 years, but not at older ages (45–70). The increased risk was attributable to an elevated risk during the postpartum period, and to a young age (≤22 years) at first birth as compared with nulliparity [83].

### 4.3. Breastfeeding

A meta-analysis including three case-control and three cohort studies suggested that breastfeeding was inversely associated with the risk of RA (OR = 0.67, 95% CI 0.5–0.9), whatever the duration [96]. Nevertheless, a dose response effect of duration of breastfeeding has been found in several studies (Table 2) [77,79,85], especially for ACPA-positive RA [83]. There were only two studies that did not find any association [14,78], and one case-control study that found an increased risk of RA associated with breastfeeding and its duration [86].

### 4.4. Benign Gynecological Diseases

Endometriosis is associated with high estrogen levels during a women’s reproductive period. A recent meta-analysis of five studies (two cross-sectional, one case-control, and two cohort studies) did not demonstrate any association between endometriosis and RA [97]. The pooled relative risk of the two prospective cohort studies was not statistically significant (RR = 1.46, 95% CI 0.70–3.03) [78,97,98]. Nevertheless, Harris et al. found a significant association between surgically confirmed endometriosis and RA (Table 2) [88].

Polycystic ovary syndrome, associated with anovulation and low serum progestogen levels, was associated with RA in a single cohort study (Table 2) [78].

### 4.5. Hormonal Treatments

Numerous studies have assessed the association between oral contraception (OC) and post-menopausal hormone therapy (PMHT) use with conflicting results, positive, negative, or null associations with risk of RA (Table 2).

A meta-analysis of 28 observational studies suggested a protective effect of oral contraception (OC) (ever versus never) in pooled case control studies (OR = 0.70, 95% CI 0.5–0.9) but not in pooled cohort studies (OR = 1.0, 95% CI 0.9–1.1). Current and past uses of OC were not associated with RA in pooled cohorts but there was a borderline inverse association in pooled case-control studies (past versus never OR = 0.70 and 95% CI 0.4–1.0, current versus never OR = 0.71 and 95% CI 0.5–1.0) [98]. Moreover, no dose-response association was found between OC use and risk of RA in this meta-analysis. These results highlighted a possible recall bias in case-control studies.

In addition, a recent case-control study reported an inverse association between OC use (ever, past, and >7 years versus never) and ACPA-positive RA (Table 2) [87], with a possible combined SE-OC effect on the risk of RA [90].

Past but not current menopausal therapy (MHT) was positively associated with RA risk in the Iowa Women’s Health Study (IWHS) and the Nurses’ Health Study (NHS) I cohorts (Table 2) as compared with never use [77,78]. However, a more recent analysis of the NHSs studies found a positive association between current MHT use (versus never HR = 1.4, 95% CI 1.1–1.9) and seropositive RA but only in the NHS I, whereas not in the NHS II or when pooling NHS I and II [89]. In the pooled cohorts, a duration of eight years and more of MHT (versus never) was associated with an increased risk of seropositive RA.

In case-control studies, MHT has not been associated with RA risk altogether, although current use of combined MHT has been inversely associated with ACPA-positive RA in menopausal women aged 50–59 with no effect of duration [99] (Table 2).

Thus, studies on OC or MHT and the risk of RA led to controversial results, potentially because of methodological issues (potential recall bias in case-control studies, insufficient accounting for confounders), changes in prescription of OCs and MHT over the past decades, and assessment of hormonal treatments as ever/never use, while analyses of durations and doses could lead to more precise estimates.

Selective estrogen receptor modulators (SERMs) and aromatase inhibitors (AI), used as a complementary treatment of breast cancers with positive estrogen receptors, reduce the endogenous production of estrogens after menopause. A study from the American national breast cancer database suggested dose-dependent associations between SERMs and AI and RA onset in women with a history of breast cancer (Table 2) [91]. The impact of AI would be stronger than tamoxifen on RA risk [92].

## 5. Diet

Many food components and beverages have been investigated in relation to RA risk in case-control and cohort studies. Several underlying mechanisms have been suggested, including the antioxidant effect of food, or the impact of diet on the gut microbiota, the involvement of which in RA pathophysiology has been suggested in different studies [100].

However, many studies regarding food have shown conflicting results. Some associations might be restricted to some populations, i.e., younger women, or ever-smokers. Table 3 summarizes results from selected cohorts and case-control studies, regarding the association between diet and the risk of RA.

### 5.1. Fish Consumption

Fish consumption has been thought to be associated with a reduced risk of RA, but different studies have led to conflicting results. Potential mechanisms involve omega-3 fatty acids, which have been suggested to lower the risk of developing ACPAs and to prevent the onset of inflammatory arthritis once ACPAs are present [127]. In a case-control study, Shapiro et al. reported a lower risk of incident RA associated with high consumption of broiled and baked fish dishes [102]. However, this association was not found with other fish dishes.

Nevertheless, no association was found in four prospective cohort studies and three other case-control studies [101,104,105,106,108,109]. In a meta-analysis including 174,701 participants, Di Giuseppe et al. reported a borderline association between fish intake and the risk of RA (≥1 serving/week as compared with <1, RR 0.71, 95% CI 0.48–1.04).

More recently, in the NHSs I and II, Sparks et al. reported an increased risk of RA associated with fish consumption among women aged 55 and over [110]. However, they identified an interaction between smoking and fish consumption in that ever smokers with frequent fish consumption had only a modestly increased risk of RA as compared with a very high risk in ever-smokers with infrequent fish intakes.

Altogether, the literature regarding a potential association between fish consumption and RA risk is limited and does not allow us to state preventive advice. Potential benefit could be restricted to some high-risk populations, such as ever-smokers.

### 5.2. Olive Oil, Fruit, and Vegetables

Olive oil and its antioxidant effect have been shown to be beneficial for different health issues, such as cardiovascular diseases and cancers. Olive oil consumption has been associated with a lower risk of RA in two case-control studies [101,103]. However, two prospective cohort studies failed to report such inverse association [104,111].

Regarding fruit and vegetable consumption, two case-control studies reported an inverse association, with high consumptions of cooked vegetables (OR 0.39 for quartile 4 as compared with quartile 1, 95% CI 0.20–0.77, *P*_trend_ = 0.001) [103], or fruit (OR 0.7 for tertile 3 versus tertile 1, 95% CI 0.4–1.3, *P*_trend_ = 0.03) [112]. However, recent case-control and cohort studies failed to find any association [104,109,111]. Thus, available evidence is insufficient to recommend fruit and vegetable consumption to reduce RA risk.

### 5.3. Meat Consumption

Although an increased intake of red meat could be associated with cancer and cardiovascular risks, there is little evidence for a role in RA risk.

Pattison et al. [112] reported the first prospective investigation of red meat and risk for inflammatory polyarthritis and concluded that higher intakes of both red meat and protein increased the risk for inflammatory polyarthritis. However, they acknowledged that it remained unclear whether the observed associations were causative or whether meat consumption was a marker for other lifestyle factors. Since then, many different prospective cohort studies have investigated meat (overall, processed meat, poultry, and red meat), and have shown no association with the risk of RA [104,105,108,111,113].

### 5.4. Coffee, Tea, and Beverages

Over the last decades, many studies have investigated a potential link between consumption of coffee, tea, and other beverages and the risk of RA.

In a cross-sectional study [114], consumption of ≥four cups of coffee per day was associated with an increased risk of RF-positive RA (RR 2.20, 95% CI 1.13–4.27). Associations were similar with decaffeinated coffee (RR 2.64, 95% CI 1.46–4.79), especially among RF-positive patients [115]. Associations remained after adjustment for smoking status. Although those results were not reproducibly found in other publications [104,116,118], a meta-analysis of five studies reported a positive association between coffee consumption and RA risk (RR 2.43, 95% CI 1.06–5.55); the association was restricted to RF-positive RA (RR 1.33, 95% CI 1.16–1.52), but not with RF-negative RA (RR 1.09, 95% CI 0.88–1.35), suggesting potentially different underlying mechanisms [117].

Regarding tea consumption, the consumption of three cups or more per day has been associated with a lower risk of RA in one prospective study (RR 0.39, 95% CI 0.16–0.95) [115], but not confirmed in other prospective studies and in a meta-analysis [115,117].

A moderate consumption of alcohol has been found inversely associated with RA in several studies [119,120,121]. In the Swedish Mammography Cohort, Di Giuseppe et al. reported a statically significant 37% decrease in the risk of RA among women who drank four or more glasses of alcohol per week as compared with women who drank one glass or less (RR 0.63, 95% CI 0.42–0.96) [119]. In the NHSs I and II, Lu et al. also reported an inverse association between moderate alcohol consumption (5–10 g/day) as compared with no use (HR 0.78, 95% CI 0.61–1.00), this association being stronger for seropositive RA cases. Those results were confirmed in a meta-analysis involving 195,095 participants including 1878 RA cases, reporting an inverse association between low to moderate alcohol consumption and RA risk (RR 0.86, 95% CI 0.78–0.94), and providing some evidence of a nonlinear inverse relationship [120]. Recently, in the Swedish Epidemiologic Investigations of RA (EIRA) involving 3353 cases and 2836 matched controls, Hedström et al. reported a dose-dependent inverse association between low and moderate alcohol and RA risk as compared with no consumption (OR 0.57, 95% CI 0.49–0.66 and OR 0.49, 95% CI 0.41–0.58, respectively) [123]. Interestingly, non-drinking and the presence of HLA-DRB1 SE interacted to increase the risk for ACPA-positive RA, independent of smoking habits. However, physicians should consider the potential risks of alcohol before providing recommendations.

Finally, some studies have suggested an increased risk of RA with the consumption of sugar-sweetened soda, sometimes limited to seropositive RA (HR 1.63, 95% CI 1.15–2.30) [122].

### 5.5. Dietary Patterns

Recently, dietary pattern analysis has emerged as an alternative approach for examining the relationship between individual food items and the risk of disease. Indeed, because of the complexity of dietary habits and the interactions among foods and nutrients, examining the overall effect of diet, using dietary patterns derived from factor or cluster analysis, or dietary quality indices, could be a more realistic approach for investigating the risk of disease [128].

The Mediterranean diet (MD), widespread in Southern European countries, mainly consists of olive oil, cereal products, fresh or dried fruit and vegetables, nuts, fish, and a moderate amount of dairy, meat, and wine. This diet has been associated with significant reductions of overall mortality, as well as cardiovascular and neoplastic diseases [129]. Four studies have investigated the association between the MD and RA risk. In a Swedish nested case-control study, Sundström et al. found no association between the MD score and RA, although there was some non-statistically significant risk reduction among smokers [108]. More recently, a case-control study from the Swedish Epidemiological Investigation of RA reported an inverse association between the MD score and RA risk (OR 0.79, 95% CI 0.65–0.96) [125]. In the NHSs I and II, Hu et al. did not find any association between the alternate MD score (which does not include dairy products) and the risk of RA. However, those results might only apply to American women, whose dietary habits could differ from those of European countries [109]. Our team investigated the association between the MD and RA risk in the E3N (Etude Epidémiologique auprès de femmes de la Mutuelle générale de l’Éducation nationale) cohort study of French women [111]. There was no association overall, but in ever-smokers, there was a significant trend towards a reduced risk of RA with a higher MD score. We hypothesized that the pro-oxidant effect of smoking could be balanced by the antioxidant effect of the MD.

Other dietary patterns have also been investigated such as diet quality, evaluated by the 2010 Alternative Healthy Index (AHAI-2010), which is a dietary quality score based on recent dietary guidelines for Americans, and is composed of 11 foods and nutrients that have been consistently inversely associated with risk of chronic diseases. In the NHS I and II, Hu et al. suggested that a long-term adherence to a healthy dietary pattern may reduce RA risk in women, particularly the risk of a seropositive RA diagnosis before the age of 55 years [124].

In addition, Sparks et al. investigated, in the same cohort, the associations among the Empirical Dietary Inflammatory Pattern (EDIP), including 18 anti- and pro-inflammatory food/beverage groups weighted by correlations with plasma inflammatory biomarkers [126]. Among women ≤55 years, increasing EDIP was associated with an increased risk of RA, and specifically seropositive RA. However, no association was found among women over 55 years old.

## 6. Conclusions

To date, smoking has been reproducibly demonstrated to trigger the emergence of RA, particularly in genetically predisposed individuals.

Regarding other inhalants, silica is the most robust non-smoking inhalant risk factor for RA with potential interaction with smoking and both would share the same pulmonary mucosal and systemic inflammation hypothesis. The literature is sparse or conflicting for other inhalants such as pesticides, other occupational inhalants, and air pollution because of difficulties in precisely measuring the level of exposure. To establish an independent relationship with RA, future studies investigating the association between inhalants and RA need to carefully account for timing of exposures and smoking duration and intensity (and not only smoking status) in the analyses.

Despite numerous studies investigating potential associations among individual reproductive factors and RA risk, the role of female hormones on the risk of RA remains unclear. Bias and methodological issues (including failure to adjust on smoking) could explain some discrepancies. Each lifetime reproductive event is associated with changes in hormonal levels, either increased (early menarche, late menopause, parity, PMH, and oral contraception use) or decreased (postpartum period, early menopause, late menarche, and anti-estrogen agent treatment). Assessing cumulative hormonal exposures, and taking into account lifetime reproductive events, may be an interesting approach to study female hormonal exposures.

There have been numerous studies that have investigated the association between diet and RA, and many of them have shown conflicting results. A high consumption of coffee seems to be associated with an increased risk of RA, and a moderate consumption of alcohol is inversely associated with the risk of RA, there is less evidence regarding other food groups. However, some associations could be restricted to some populations (≤55 year-old women, ever-smokers) or be limited to seropositive RA. Nevertheless, studying associations among RA and some dietary patterns, such as inflammatory dietary index, a Mediterranean diet, or diet quality indices, might be more accurate, and some associations among those patterns and RA risk have been found. These results could be used for individuals at high risk of developing RA (i.e., RA relatives or subjects with ACPA positivity) who could modify their diets in addition to correcting major risk factors such as smoking.

## Figures and Tables

**Table 1:** Selected case-control and cohort studies on inhaled exposures associated with risk of rheumatoid arthritis.

Exposures [ref]	Study Design	Population, Occupations	Sample Size (Cases/Controls, Cohort)	Adjusted* HR/OR/RR (95% CI) for RA Comments
**SILICA**				
Klockars, 1987 [42]	Finish retrospective cohort	Male granite workers, 15–72 years	1026 including 35 RA	For dust concentration and disability pension for RA: **RR = 5.08 (3.3–7.7)** No smoking adjustment
Turner, 2000 [43]	British case-control	Pottery, sandstone, and refractory material workers born between 1916 and 1945	58/232	Duration of silica exposure per 10 years, OR = 0.31 (0.16–0.61) Cumulative exposure/1000 (µg/m^3^ per year), OR = 0.80 (0.64–1.02)
Olsson, 2004 [44]	Swedish case-control RA cases from TIRA	Men, 16–75 years, stone and/or silica dust	176/630	OR = 1.8 (0.6–5.5) vs. never
Stolt, 2005 [45]	Swedish case-control (EIRA study)	Men, 18–70 years, Stone dust, rock drilling, or stone crushing	276/276	**OR = 2.2 (1.2–3.9)** versus unexposed to silica **OR = 3.0 (1.2–7.6)** for rock drilling or stone crushing
Stolt, 2010 [46]	Swedish case-control (EIRA study)	Men, 18–70 years, Stone dust, rock drilling or stone crushing	577/659	All RA, OR = 1.39 (0.98–1.96) **ACPA+ RA, OR = 1.67 (1.13–2.48)** ACPA- RA, OR = 0.98 (0.57–-1.66) **Rock drilling, ACPA-positive RA, OR = 2.34 (1.17–4.68)** **Interaction between smoking and silica**
Yahya, 2014 [47]	Malaysian case-control (MyEIRA study)	Men, 18–70 years Stone dust, rock drilling or stone crushing	149/213	All RA, OR = 2.0 (0.9–4.6) **ACPA+ RA, OR = 2.4 (1.0–5.6)** **ACPA+ RA among smokers, OR = 7.5 (2.3–24.2)**
Blanc, 2015 [48]	Swedish construction industry retrospective cohort	Male construction workers, 30–84 years	240,983 including 713 RA	**RR = 1.33 (1.1–1.6) versus unexposed** in overall population **RR = 1.36 (1.1–1.7) among smokers** No association among never smokers
Ilar, 2019 [49]	Swedish case-control (EIRA and national register)	Male and female workers, ≥18 years	11,285/115,249	**All RA, OR = 1.3 (1.2–1.5)** **Seropositive RA, OR = 1.4 (1.2–1.5)** Seronegative RA, OR = 1.2 (1.0–1.4)
Schmajuk, 2019 [50]	US random digit dialled telephone survey	Men, work-related silica exposure	973 men	**Silica vs. never, OR = 2.1 (1.1–3.9)** No interaction effect with smoking
**OTHER INORGANIC DUSTS**				
Blanc, 2015 [48]	Swedish construction industry retrospective cohort	Male construction workers, 30–84 years	240,983 including 713 RA	**RR = 1.32 (1.1–1.6) versus unexposed** in overall population **RR = 1.42 (1.2–1.7) among smokers** No association among never smokers
**TEXTILE DUST**				
Too, 2016 [51]	Malaysian case-control (MyEIRA study)	Women, 18–70 years	910/910 (≥96% never smokers)	**All RA, OR = 2.8 (1.6–5.2)** **ACPA+ RA, OR = 2.5 (1.3–4.8)** **ACPA– RA, OR = 3.5 (1.7–7.0)** Interaction with SE alleles regarding the risk of ACPA-positive RA (OR for double exposed 39.1, 95% CI 5.1–297.5, attributable proportion due to interaction 0.8, 95% CI 0.5–1.2).
**MINERAL OIL**				
Sverdrup, 2005 [52]	Swedish case control	18–70 years, occupational exposure to any mineral oil, cutting oil, motor oil, form oil, hydraulic oil, and asphalt	1419/1674	Any mineral oil: All RA, RR = 1.3 (1.0–1.7); RF+, RR = 1.4 (1.0–2.0); and RF-, RR = 1.0 (0.6–1.5) Hydraulic oil: All RA, RR = 1.4 (1.0–2.0); RF+, RR = 1.5 (1.0–2.3); and RF-, RR = 1.2 (0.7–2.1) No association with cutting oil, motor oil, form oil, and asphalt
**STYRENE**				
Boudigaard, 2020 [53]	Danish national pension fund register (1979–2012)	Plastics industry	72,212 including 527 RA (83% men)	Cumulative styrene exposure (mg/m^2^ per year): For high exposure (≥68 vs. ≤17), RR = 1.95 (1.05–6.61) for seropositive RA among women
**PESTICIDE/INSECTICIDES**				
Meyer, 2017 [54]	Case control in Agricultural Health Study (Iowa and North Carolina)	Male farmers (pesticide applicators)	220/26,134	**Fonofos (organophosphate vs. never), OR = 1.70 (1.22–2.37)** **Carbaryl (carbamate vs. never), OR = 1.51 (1.03–2.23)** **Chlorimuron ethyl vs. never), OR = 1.45 (1.01–2.07)** Exposure–response trends were observed for lifetime days of use of atrazine and toxaphene
Parks, 2016 [55]	Case control in Agricultural Health Study spouses	Farmers’ spouses	132/24,018	Any use of pesticide vs. never, OR = 1.4 (1.0–1.6) Of the 15 pesticides examined: **Maneb/mancozeb vs. never, OR = 3.3 (1.5–7.1);** glyphosate vs. never, OR = 1.4 (1.0–2.1); **application of chemical fertilizers, OR = 1.7 (1.1–2.7);** **cleaning with solvents, OR = 1.6 (1.1–2.4)**
De Roos, 2005 [56]	Nested case-control in Agricultural Health Study	Women	135/675	Applying pesticides, OR = 1.8 (0.6–5.0) Welding fumes, OR = 1.8 (0.6–5.6)
Parks, 2018 [57]	Case control in U.S. Sister Study	Women, 35–74 years Exposition to pesticide during childhood	424/48,919	Women reporting childhood-only farm residence with: **Personal use of pesticides on crops, OR = 1.8 (1.1–2.9);** contact with livestock and pesticide use. **OR = 2.0 (1.2–3.3);** pesticide use on crops and animals, **OR = 2.0 (1.2–3.2)**
**OTHER INHALANT-RELATED OCCUPATIONS**				
Parks, 2019 [58]	Cohort, Agricultural Health Study	Framers and spouses, no pesticide agricultural exposures	49,406 including 478 RA	**Regularly applying chemical fertilizers, HR = 1.50 (1.11–2.02)** **Non-gasoline solvent use, HR = 1.40 (1.09–1.80)** **Other cleaning solvent use, HR = 1.40 (1.09–1.80)**
De Roos, 2005 [56]	Nested case-control in Agricultural Health Study	Women, farmers and welders	135/675	Welding fumes, OR = 1.8 (0.6–5.6)
Ilar, 2018 [59]	Swedish case-control (EIRA study)	Men and women, 18–70 years, occupation related to potential noxious airborne agents	3522/5580	For ACPA+ RA among men: **Bricklayers/concrete workers, OR = 2.9 (1.4–5.7);** **material handling operators, OR = 2.4 (1.3–4.4);** **electrical and electronic workers, OR = 2.1 (1.1–3.8)** No occupation related to airborne agent was significantly associated with RA among women
Olsson, 2000 [60]	Swedish case-control (monocentric)	Men and women, 25–75 years, various noxious airborne agents	422/859	For men: Farmers, OR = 1.8 (1.0–3.5); **asphalters, OR = 14.0 (1.2–16.2);** textile workers, OR = 2.0 (0.3–16.2)
Olsson, 2004 [44]	Swedish case-control RA cases from TIRA (acronym for ‘‘early intervention in rheumatoid arthritis’’) cohort	Men and women, 16–75 years, various noxious airborne agents	715 incident and prevalent RA/2204 293 incident RA/1346	Only in men (vs. no exposure): **Conductors, freight, and transport workers, OR = 4.7 (1.4–16.3);** **farmers and farm workers; OR = 2.2 (1.3–3.5);** **asbestos, OR = 2.5 (1.0–6.8)** No association with mineral oil and pesticides. Nonlinear significant association with duration of exposure to fertilizers, crops, and/or forage
Cappelletti, 2016 [61]	Italian retrospective cohort (1979–2009)	Men, steel workers exposed to foundry dust	331	**RR = 6.17 (2.0–19.0)** No adjustment for smoking
Lundberg, 1994 [62]	Swedish retrospective cohort	Men and women born between 1905 and 1945, same occupation for at least 10 years	375,035 men and 140,139 women, 1525 RA	Only in men: Farmers, RR = 1.3 (1.0–1.6); **spray painters and lacquer workers, RR = 2.4 (1.1–5.4);** **concrete and construction workers, RR = 1.4 (1.1–2.0);** **organic solvent (substantial use), RR = 1.2 (1.0–1.6);** No association with mineral oil, pesticides, asbestos, petrol No adjustment for smoking
Noonan, 2006 [63]	Libyan nested case-control	Vermiculite/asbestos exposure	7307 residents, 129 RA	Military asbestos exposure (vs. never), OR = 2.11 (1.04–4.30) **Dust of vermiculite exposure (vs. never); OR = 1.65 (1.14–2.39)** **≥65 Years vermiculite mining company workers, OR = 3.23 (1.31–7.96)**
Jones, 2012 [64]	U.S. military prospective cohort	Smoke from open-air burn pits	18,848 with 234 RA	Three Mile from a burn pit deployment (vs. >3 miles), OR = 1.17 (0.83–1.64) No association with number of exposed days
Schmajuk, 2019 [50]	U.S. random digit dialed telephone survey	Men, coal miners	973 men	**Coal mining work (vs. never), OR = 3.6 (2.1–6.2)** No interaction effect with smoking
**TRAFFIC POLLUTION**				
Hart, 2009 [65]	U.S. cohort (NHS I)	Nurses, 30–55 years, distance to a major road as marker of traffic pollution exposure	90,297 including 687 RA	Living distance to road <50 m vs. ≥200 m: All RA, HR = 1.31 (0.98–1.74); **RF + RA, HR = 1.44 (1.0–2.07);** RF – RA, HR = 1.15 (0.73–1.83) **Among non-smokers, HR = 1.62 (1.04–2.52)**
De Roos, 2014 [66]	Canadian nested case-control (British Columbia Health Insurance System)	Residential distance from highway or major road	1911/19,066	**Distance from highway (residence ≤50 m vs. >150 m away), OR = 1.37 (1.11–1.68)** Distance from major road (residence ≤50 m vs. >150 m away), OR = 1.02 (0.92–1.14)
**AMBIENT AIR POLLUTION**				
Shin, 2019 [67]	Korean nested case-control (2002–2014)	>20 years, one-year average concentrations of air pollution predicted by residential addresses	444/1776	Ozone (vs. <37.66 ppb): [37.66–39.70], OR = 1.17 (0.86–1.59); **[39.70–42.11], OR = 1.45 (1.08–1.96);** **≥42.11, OR = 1.35 (1.00–1.83)** Carbon monoxide (vs. <465.34 ppb): [465.34–50.7], OR = 1.74 (1.24–2.44); **[509.7–552.25], OR = 1.83 (1.24–2.70);** **≥552.25: OR = 1.83 (1.11–3.01)** No association with PM_10_, SO2, NO2
Hart, 2013 [68]	U.S. cohort (NHS)	Nurses, 30–55 years, outdoor levels of PM10 and PM2.5, SO2 and NO2	111,425 including 858 RA	HR per IQR range: NO_2_ (15.3 µg/m^3^), HR = 0.92 (0.85–1.0); PM_2.5_ (5 µg/m^3^), HR = 0.94 (0.86–1.04); PM_10_ (7 µg/m^3^), HR = 0.92 (0.85–0.99); SO_2_ (14 µg/m^3^), HR = 0.99 (0.90–1.09)
Hart, 2013 [69]	Case-control (Swedish EIRA)	18–70 years, levels of PM_10_, SO_2_ and NO_2_, from traffic and home heating	1497/2536	OR per IQR increase over average: NO_2_ (9 µg/m^3^), OR = 0.98 (0.90–1.07); PM_10_ (2 µg/m^3^), OR = 0.96 (0.88–1.04); SO_2_ (8 µg/m^3^), OR = 1.01 (0.93–1.09)
De Roos, 2014 [66]	Canadian nested case-control (British Columbia Health Insurance System)	Monthly air pollutant levels averaged over residences: PM_10_, PM2.5, SO_2_ and NO_2_, ozone, CO, black carbon	1911/19066	OR per IQR increase: NO_2_ (6.3 µg/m^3^), OR = 0.90 (0.85–0.96); PM_2.5_ (2.7 µg/m^3^), OR = 0.92 (0.87–0.98); PM_10_ (0.87 µg/m^3^), OR = 0.91 (0.86–0.96); SO_2_ (3.1 µg/m^3^), OR = 0.88 (0.82–0.93) **Ground-level ozone** (8.6 µg/m^3^), **OR = 1.26 (1.18–1.36)**
Chang, 2016 [70]	Cohort from Taiwan (Health Assurance Database)	Yearly average air pollutant concentrations of NO_2_ and PM_2.5_,	For NO_2_ exposure: 247,419 including 376 RA For PM_2.5_ exposure: 244,413 with 236 RA	HR per pollutant levels **NO_2_ (vs. <66.21 ppm):** [66.2–86.10], HR = 1.12 (0.83–1.52); **[86.10–99.88], HR = 1.53 (1.12–2.90);** **>99.88, HR = 1.52 (1.11-2.08)** PM_2.5_ (vs.<10.7 µg/m^3^): [10.7–12.16], HR = 1.22 (0.85–1.74); [12.16–15.05], HR = 1.15 (0.82–1.62); >15.05, HR = 0.79 (0.53–1.16) No adjustment on smoking

NHS, Nurses’ Health Study; RR, relative risk; OR, odds ratio; HR, hazard ratio; 95% CI, 95% confidence interval; RF, rheumatoid factor; ACPA, anti-citrullinated peptide antibodies; SE, shared epitope; PM, fine particulate matter; NO2, nitrogen dioxide; SO2, sulfur dioxide; CO, carbon monoxide. * Adjusted for smoking status (at least).

**Table 2:** Selected case-control and cohort studies on reproductive factors and the risk for rheumatoid arthritis.

Reproductive Factors [ref]	Study Design	Sample Size (Cases/Controls, Cohort)	Adjusted HR/OR/RR (95% CI) for All RA (Unless Specified Otherwise) Comments
**Menarche and menstrual periods**			
Karlson, 2004 [77]	NHS I (1976–2002)	121,700 women including 674 RA	**Early menarche** (≤10 years vs.12 years), **RR = 1.6 (1.1–2.4) for seropositive RA** Menstrual periods (very irregular vs. very regular), RR = 1.4 (1.0–2.0)
Pedersen, 2006 [14]	Danish case-control	515/769	**Late menarche (≥15 vs. ≤12 years), OR = 1.87 (1.23–2.85)** No association with number of live-birth children, miscarriages, breast feeding, age at menopause
Merlino. 2003 [78]	IWHS	31,336 women age 55–69 years including 158 RA	Age at menarche (>14 vs. <11 years), HR = 0.9 (0.3–2.4)
Pikwer, 2012 [79]	Nested case-control community–based health survey	136/544	**Early menarche** (<12 vs. ≥12 years), **OR = 0.29 (0.06–0.77) for all RA** **Seropositive RA, OR = 0.18 (0.04–0.81)** Seronegative RA, OR = 0.28 (0.32–2.52)
**Postpartum and Parity**			
Jorgensen, 2010 [80]	Cohort (National Danish Cohort)	2,140,056 women born in 1935–89 including 1648 RA	**Two child mothers (vs. 1), RR = 0.84 (0.78–0.90)** **Three child mothers (vs. 1), RR = 0.83 (0.77–0.91)** **Age at the first child ≥30 years (vs. 20–24 years), RR = 0.76 (0.68–0.85)**
Guthrie, 2010 [81]	U.S. case-control	310/1418	**Parous (vs. nulliparous). RR = 0.61 (0.4–0.8) for all ages** Within age groups <35 years, RR = 0.54 (0.33 – 0.90) and 35–44 years RR = 0.52 (0.30–0.90) No association after 45 years No association with age at the first birth and number of births
Silman, 1992 [82]	UK case-control	88/144	RA onset during pregnancy, OR = 0.30 (0.04–2.6) RA onset during the first 3 months postpartum, OR = 5.6 (1.8–17.6) RA onset during 4–12 months postpartum, OR = 2.6 (0.8–7.9)
Orellana, 2014 [83]	Case-control (EIRA)	603/906 women aged 18–44 years	**In ACPA-negative RA and in the age-group 18–44 years:** **Parity (yes vs. no), OR = 2.1 (1.4–3.2);** **delivery during the year of the symptom onset (vs. nulliparous), OR = 2.6 (1.4–4.8);** **age at the first birth (≤22 years vs. nulliparous); OR = 2.5 (1.5–4.1)**
Peschken, 2012 [84]	Case-control (North American Natives)	168/400	**12 months postpartum, OR = 3.8 (1.5–9.9)** **≥6 births (vs. 1-2 births), OR = 0.43 (0.21–0.87)**
Merlino, 2003 [78]	IWHS	31,336 women age 55–69 years including 158 RA	Parous (vs. nulliparous), HR = 1.14 (0.63–2.05) >Five pregnancies (vs. 0), HR = 0.76 (0.34–1.71) Age at the first pregnancy >25 vs. <20 years, HR = 0.65 (0.39–1.08) Age at the last pregnancy >34 vs. <25 years, HR = 0.64 (0.38–1.09) No association with the number of miscarriages and stillbirths
Karlson, 2004 [77]	NHS I (1976–2002)	121,700 women including 674 RA	Nulliparous vs. parous, RR = 1.3 (0.9–1.9) Age at the first birth (>29 vs. <20 years), RR = 0.8 (0.5–1.1) Parity (≥4 vs. 0), RR = 0.8 (0.5–1.2)
Pikwer 2012 [79]	Nested case-control community-based health survey	136/544	Give birth to ≥1 child (vs. nulliparous): All RA, OR = 0.75 (0.45–1.24); seropositive RA, OR = 0.84 (0.44–1.60); seronegative RA, OR = 0.64 (0.27–1.52)
Adab, 2014 [85]	Chinese nested case-control (Guangzhou Biobank Cohort)	7349 women ≥50 years	Parity (increasing number of live births): OR = 1.0 (0.92–1.10)
**Breast Feeding**			
Karlson, 2004 [77]	NHS I (1976–2002)	121,700 women including 674 RA	**Duration ≥24 months** (vs. no breast–feeding): **All RA, RR = 0.5 (0.3–0.8);** seropositive RA, RR = 0.6 (0.3–1.1)
Merlino, 2003 [78]	IWHS	31,336 women age 55–69 years including 158 RA	Number of children breastfed (>2 vs. 0), HR = 0.64 (0.37–1.09) No association with drug to stop lactation
Pikwer, 2012 [79]	Nested case-control community-based health survey	136/544	**Breastfeeding >13 months** (vs. no breastfeeding): **All RA, OR = 0.46 (0.24–0.91);** seropositive RA, OR = 0.61 (0.28–1.36); seronegative RA, OR = 0.30 (0.08–1.17)
Berglin, 2010 [86]	Nested case-control (Medical Biobank of northern Sweden)	70/280	**Ever vs. never, OR = 4.8 (1.43–15.87)** **≥17 months (*vs.* 0–3 months), OR = 5.7 (1.83–17.95)** No association with age at the first pregnancy, age at menarche, age at menopause, miscarriages, number of biological children
Adab, 2014 [85]	Nested case-control	7349 Chinese women ≥50 years	**Ever vs. never, OR = 0.45 (0.23–0.88);** **>36 months vs. never, OR = 0.48 (0.27–0.86)**
Orellana, 2017 [87]	Case-control (EIRA)	2641/4251	**In all RA (≥13 months vs. ≤6 months), OR = 0.77 (0.63–0.94)** **In ACPA-positive RA (≥13 months vs. ≤6 months), OR = 0.74 (0.59–0.93)** No association with ACPA-negative RA
**Endometriosis**			
Harris, 2016 [88]	NHS II	116,430 women including 390 RA	**Laparoscopically confirmed endometriosis vs. Never, HR = 1.41 (1.05–1.89)**
Merlino, 2003 [78]	IWHS	31,336 women aged 55–69 years including 158 RA	Ever vs. never, HR = 1.72 (0.93–3.18)
**Polycystic Ovary Syndrome**			
Merlino, 2003 [78]	IWHS	31,336 women aged 55–69 years including 158 RA	**Self-reported ever vs. never, HR = 2.58 (1.06–6.30)**
**Menopausal Factors**			
Bengtsson, 2017 [89]	NHS I (1976–2010) and NHS II (1989–2011)	120,700 nurses aged 30–55 years 116,430 nurses aged 25–42 years	For seronegative RA (RF and/or ACPA): **Post-menopause, HR = 2.1 (95% CI 1.4–3.0);** **early age at natural menopause (≤44 years vs. premenopause), HR = 2.4 (95% CI 1.5–4.0)** Other menopausal factors (type, number of ovulatory years) were not associated with the 2 subsets of RA
Merlino, 2003 [78]	IWHS	31,336 women age 55–69 years including 158 RA	**Age at menopause > 51 years (vs. <45), HR = 0.64 (0.41–1.0)** No association with the number of ovulatory years
Pikwer, 2012 [79]	Nested case control community-based health survey	136/544	**Early age at menopause (≤45 vs. >45 years):** **All RA, OR = 1.92 (1.02–3.67);** seropositive RA, OR = 1.98 (0.91–4.31); **seronegative RA, OR = 5.00 (1.72–14.51)**
**Oral contraception (OC)**			
Karlson, 2004 [77]	NHS I (1976–2002)	121,700 women including 674 RA	Ever vs. never, RR = 1.1 (0.9–1.3) Duration (≥5 vs. 0 years), RR = 1.0 (0.8–1.3)
Merlino, 2003 [78]	IWHS	31,336 women age 55–69 years including 158 RA	Ever vs. never, HR = 1.0 (0.7–1.5)
Berglin, 2010 [86]	Nested case-control	70/280	**Duration >7 years (vs. never use), OR = 0.37 (0.15–0.93)**
Adab, 2014 [85]	Nested case-control	7349 Chinese women ≥50 years	Ever vs. never, OR = 1.18 (0.84–1.67) Duration ≥5 years vs. 0, OR = 0.89 (0.41–1.92)
Orellana, 2017 [87]	Case-control (EIRA)	2641/4251	**In all RA:** **Ever vs. never, OR = 0.87 (0.78–0.97);** **past vs. never, OR = 0.87 (0.78–0.98);** **ever and duration >7 years vs. never, OR = 0.81 (0.71–0.92);** **past and duration >7 years vs. never, OR = 0.81 (0.71–0.93)** **In ACPA-positive RA:** **Ever vs. never, OR = 0.84 (0.74–0.96);** **past vs. never, OR = 0.83 (0.73–0.95);** **ever and duration >7 years vs. never, OR = 0.80 (0.69–0.93);** **past and duration >7 years vs. never, OR = 0.80 (0.68–0.93)**
Pedersen, 2006 [14] Pedersen, 2007 [90]	Danish case-control	515/769	All RA (ever vs. never), OR = 1.24 (0.9–1.7) ACPA-positive RA (ever vs. never), OR = 1.65 (1.06–2.57) **ACPA-negative RA (ever vs. never), OR = 1.20 (1.68–2.07)** ACPA-positive RA, SE homozygotes + OC use, OR = 44.6 (15.2–131) as compared with noncarriers SE and never use of OC.
**Post-Menopausal Hormonal Treatment (PMHT)**			
Karlson, 2004 [77]	NHS I (1976–2002)	121,700 women including 674 RA	**Past vs. never, RR = 1.3 (1.0–1.6)** Current vs. never, RR = 1.0 (0.8–1.3)
Bengtsson, 2017 [89]	NHS I (1976–2010) and NHS II (1989–2011)	120,700 (nurses 30–55 years) 116,430 (nurses 25–42 years)	**PMHT duration ≥8 years (vs. never), HR = 1.4 (1.1–1.9) for seropositive RA** **NHS I, current use, HR = 1.4 (1.1–1.9) for seropositive RA** No association with PMH use (ever, past or current vs. never) and age at PMHT initiation in pooled NHS I and II
Merlino, 2003 [78]	IWHS	31,336 women age 55–69 years including 158 RA	**Former vs. never, HR = 1.47 (1.04–2.06)** Current vs. never, HR = 1.02 (0.61–1.72) No association with duration
**Post–Menopausal Hormonal Treatment (PMHT)**			
Orellana, 2015 [87]	Case-control (EIRA)	567/935 post-menopausal women	In ACPA-positive RA **Current use of PMH (vs. never) by age-groups:** **50–59 years, OR = 0.3 (0.1–0.8);** 60–70 years, OR = 0.8 (0.4–1.4); Oestrogen only, OR = 0.8 (0.5–1.6); **Oestrogen + progestogens, OR = 0.3 (0.1–0.7)** No association with duration of PMHT or ACPA-negative RA
Pedersen, 2006 [14]	Danish case-control	515/769	No association with PMHT (ever vs. never)
**Anti-Estrogen Agents (SERMs or AI)**			
Cheg, 2015 [91]	Nested case-control (U.S. national database on breast cancer)	238,880 women with breast cancer	**SERMs:** **<12 months (vs. no SERMs), OR = 1.3 (1.1–1.4);** **≥12 months (vs. no SERMs), OR = 2.4 (1.9–3.0)** **AI:** **>12 months (vs. no AI), OR = 1.3 (1.2–1.4);** **≥12 months (vs. no AI), OR = 1.9 (1.6–2.1)**
Caprioli, 2017 [92]	Italian cohort	10,493 women with breast cancer and treatment with AI or tamoxifen	**AI (vs. tamoxifen), HR = 1.62 (1.03–2.56)** **Anastrozole (vs. tamoxifen), HR = 1.75 (1.07–2.86)**

NHS, Nurses’ Health Study (USA); IWHS, Iowa Women’s Health Study; EIRA, Swedish Epidemiological Investigation of RA; RR, relative risk; OR, odds ratio; HR, hazard ratio; 95% CI, 95% confidence interval; RF, rheumatoid factor; ACPA, anti-citrullinated peptide antibodies; OC, oral contraception; PMHT, post-menopausal hormonal treatment; SERM, selective oestrogen receptor modulator; AI, aromatase inhibitor; SE, shared epitope.

**Table 3:** Selected case-control and cohort studies on diet and the risk for rheumatoid arthritis.

Food Component [Ref]	Study Design	Sample Size (Cases/Controls, Cohort)	Adjusted HR/OR/RR (95% CI) for All RA (Unless Specified Otherwise) Comments
**Fish Consumption**			
Linos, 1991 [101]	Case-control	168/137	Fish, for ≥12 servings/month, RR 0.37 (0.13–1.05)
Shapiro, 1996 [102]	Case-control	324/1245	Fish, for ≥2 servings/day versus <1 serving/day: Fried fish, OR 1.27 (0.84–1.92); tuna, tuna salad, tuna casserole, OR 1.19 (0.83–1.72); shellfish, OR 0.95 (0.47–1.94); **broiled or baked fish, OR 0.57 (0.35–0.93);** all fish items, OR 0.92 (0.67–1.25) Omega-3-fatty acids, quartile 4 versus quartile 1 of consumption (≤0.2 g/day): 0.2–0.5 g/day, OR 1.10 (0.78–1.56); >0.5–0.9 g/day, OR 1.01 (0.70–1.44); >0.9–1.6 g/day, OR 1.02 (0.67–1.55); >1.6 g/day, OR 0.77 (0.46–1.27) No adjustment for smoking status
Linos, 1999 [103]	Case-control	145/100	Fish, for quartile 4 (high) versus quartile 1 (low), OR 0.95 (0.46–1.96), *P*_trend_ = 0.65
Pedersen, 2005 [104]	Cohort study Danish National Patient Registry	57,053 including 69 incident RA	All types, per 30 g/day, RR 0.91 (0.68–1.23) Fish, lean, per 30 g/day, RR 0.83 (0.47–1.46) Fish, medium fat, per 30 g/day, RR 2.33 (0.99–4.30) Fish, fat, per 30 g/day, RR 0.62 (0.32–1.22)
Benito-Garcia, 2007 [105]	Cohort study NHS I	82,063 including 546 cases	Fish, quintile 5 versus quintile 1, HR 0.96 (0.72–1.26), *P*_trend_ = 0.088
Di Giuseppe, 2013 [106]	Cohort study Swedish Mammography Cohort	32,232 women including 205 RA cases	Fish, ≥1 serving/week versus <1, RR 0.71 (0.48–1.04)
Di Giuseppe, 2014 [107]	Meta-analysis	174,701 including 3346 RA cases	For every 1 serving per week increase in fish consumption, RR 0.96 (0.91–1.01)
Sundström, 2015 [108]	Nested case-control	386/1886	Fish, for tertile 3 versus tertile 1: All RA, OR 0.94 (0.71–1.26); anti-CCP-positive RA, OR 0.94 (0.67–1.31); RF-positive RA, OR 0.98 (0.72–1.34)
Hu, 2015 [109]	Cohort study NHS I and II	174,638 women including 913 incident RA cases	Fish, quartile 4 versus quartile 1, HR 1.15 (0.95–1.40), *P*_trend_ = 0.12
Sparks, 2019 [110]	Cohort study (NHS II)	166,013 women including 1080 incident RA cases	For ≥4 fish servings/week versus none to <1/month, *p* for trend: All RA, HR 0.93 (0.67–1.28), *P*_trend_ = 0.42; seropositive RA, HR 0.88 (0.58–1.33), *P*_trend_ = 0.66; seronegative RA, HR 1.01 (0.59–1.71), *P*_trend_ = 0.45; all RA among ≤55 years, HR 0.72 (0.47–1.11), *P*_trend_ = 0.29; **all RA among >55 years, HR 1.32 (0.76–2.27), *P*_trend_ = 0.037** **Interaction between fish intake and smoking among all RA <55 years** Compared with never-smokers with frequent fish intake: - ever-smokers with frequent fish intake, HR 1.29 (1.07–1.57); - ever-smokers with infrequent fish intake, HR 2.59 (1.65–4.06)
Nguyen, 2020 [111]	Cohort study E3N	62,629 women including 480 incident RA cases	Fish consumption, for tertile 3 versus tertile 1, HR 0.99 (0.80–1.22), *P*_trend_ = 0.65
**Olive Oil**			
Linos, 1991 [101]	Case-control	168/137	**>30 mL/month versus <6, RR 0.26 (0.07–0.98), *P*_trend_ = 0.01**
Linos 1999 [103]	Case-control	145/100	**For quartile 4 (high) versus quartile 1 (low consumption), OR 0.39 (0.19–0.82), *P*_trend_ = 0.03**
Pedersen, 2005 [104]	Cohort study Danish National Patient Registry	57,053 including 69 incident RA	Olive oil, per g/day, HR 1.00 (0.92–1.08)
**Fruits and Vegetables**			
Linos,1999 [103]	Case-control	145/100	Raw vegetables For quartile 4 (high) versus quartile 1 (low consumption), OR 0.85 (0.44–1.67), *P*_trend_ = 0.78 Cooked vegetables** For quartile 4 (high) compared with quartile 1 (low consumption), OR 0.39 (0.20–0.77), *P*_trend_ = 0.001**
Pattison, 2004 [112]	Nested case-control EPIC Norfolk	88/176	**-Fruit, tertile 1 versus tertile 3, OR 2.1 (1.1–4.2), *P*_trend_ = 0.03**
Pedersen, 2005 [104]	Cohort study Danish National Patient Registry	57,053 including 69 incident RA	All vegetables and vegetable juices, per 100 g/day, IRR 0.95 (0.75–1.19) All vegetable, and fruits and juices, per 100 g/day, IRR 0.95 (0.85–1.05) All fruits and fruit juices, per 100 g/day, IRR 0.89 (0.75–1.05) Citrus fruits, per 100 g/day, IRR 1.02 (0.64–1.64)
Sundström, 2015 [108]	Nested case-control	386/1886	Vegetables, highest tertile versus lowest, OR 0.79 (0.57–1.10) Fruit, highest tertile versus lowest, OR 0.88 (0.66–1.17)
Hu, 2015 [109]	Cohort study NHS I and II	174,638 women including 913 incident RA cases	Vegetables, quartile 4 versus quartile 1, HR 1.13 (0.92–1.38), *P*_trend_ = 0.15 Fruit, quartile 4 versus quartile 1, HR 0.95 (0.77–1.17), *P*_trend_ = 0.86
Nguyen, 2020 [111]	Cohort study E3N	62,629 women including 480 incident RA cases	Raw vegetables, for tertile 3 versus tertile 1, HR 0.95 (0.76–1.20), *P*_trend_ = 0.57
**Meat Consumption**			
Pattison, 2004 [112]	Nested case-control EPIC Norfolk	88/176	Red meat, tertile 3 versus tertile 1, OR 1.9 (0.9–4.0), *P*_trend_ = 0.08 Meat products, tertile 3 versus tertile 1, OR 1.4 (0.7–2.7), *P*_trend_ = 0.4 **Total meat, tertile 3 versus tertile 1, OR 2.3 (1.19–4.9), *P*_trend_ = 0.03**
Pedersen, 2005 [104]	Cohort study Danish National Patient Registry	57,053 including 69 incident RA	Red meat, fish, poultry, processed meat, per 100 g/day, IRR 1.16 (0.83–1.62) Red meat, per 100 g/day, IRR 1.36 (0.75–2.47)
Benito-Garcia, 2007 [105]	Cohort study NHS I	82,063 including 546 cases	Total meat, quintile 5 versus quintile 1, HR 0.91 (0.67–1.23), *P*_trend_ = 0.55 Red meat, quintile 5 versus quintile 1, HR 0.86 (0.64–1.16), *P*_trend_ = 0.35 Poultry, quintile 5 versus quintile 1, HR 1.17 (0.88–1.55), *P*_trend_ = 0.06
Sundström, 2019 [113]	Cohort study (Swedish Mammography Cohort)	35,600 women including 368 incident RA	Meat, overall, for >10 servings/week versus ≤4 servings/week, HR 1.08 (0.77–1.53) Read meat, for >10 servings/week versus ≤4 servings/week, HR 1.08 (0.77–1.50) Processed meat, for >6 servings/week versus ≤1 servings/week, HR 0.83 (0.59–1.22) Poultry, for >1 servings/week versus 0 servings/week, HR 0.88 (0.60–1.31)
Hu, 2015 [109]	Cohort study NHS I and 2	174,638 women including 913 incident RA cases	Red/processed meat, quartile 4 versus quartile 1, HR 1.10 (0.85–1.43), *P*_trend_ = 0.51
Nguyen, 2020 [111]	Cohort study E3N	62,629 women including 480 incident RA cases	Meat, for tertile 3 versus tertile 1, HR 1.03 (0.82–1.30), *P*_trend_ = 0.80
**Coffee, Decaffeinated Coffee, Tea**			
Heloiövaara, 2000 [114]	Cross sectional	126/6809	Coffee consumption: All RA, *P*_trend_ = 0.04; RF-positive RA, *P*_trend_ = 0.02; RF-negative RA, *P*_trend_ = 0.88
Mikuls, 2002 [115]	Cohort study Iowa Women’s Health Study	31,336 women including 158 incident RA cases	Total coffee, for ≥4 cups/day versus none, RR 1.56 (0.80–3.06), P_trend_ = 0.21 Caffeinated coffee, for ≥4 cups/day versus none, RR 0.98 (0.60–1.61), P_trend_ = 0.46 **Decaffeinated coffee, for ≥4 cups/day versus none, RR 2.44 (1.52–3.89), P_trend_ = 0.003** **RF-positive RA, RR 2.64 (1.46–4.79), P_trend_ = 0.006** RF-negative RA, RR 1.63 (0.64–4.12), P_trend_ = 0.68 Tea, for ≥3 cups/day versus none, RR 0.35 (0.13–0.97), P_trend_ = 0.50 RF-positive RA, RR 0.24, (0.06–1.09), P_trend_ = 0.43 RF-negative RA, RR 0.67 (0.15–2.91), P_trend_ = 0.86
Karlson, 2003 [116]	Cohort study NHS I	121,703 women including 480 incident RA cases	Caffeinated coffee, for ≥4 cups/day versus 0, RR 1.3 (0.9–1.8) Decaffeinated coffee, for ≥4 cups/day versus 0, RR 1.2 (0.6–2.4) Total coffee, for ≥4 cups/day versus 0, RR 1.3 (1.0–1.8)
Pattison, 2004 [112]	Nested case-control EPIC Norfolk	88/176	Caffeinated coffee, tertile 3 versus tertile 1, OR 1.1 (0.6–2.2), *P*_trend_ = 0.8 Tea, tertile 3 versus tertile 1, OR 1.5 (0.8–2.8), *P*_trend_ = 0.2
Pedersen, 2005 [104]	Cohort study Danish National Patient Registry	57,053 including 69 incident RA	Coffee, per 200 g/day, IRR 1.10 (0.99–1.21)
Lee, 2014 [117]	Meta-analysis	1279 RA cases and 133,622 non cases	Coffee consumption RR, 2.43 (1.06–5.55) Tea consumption, RR = 0.88 (0.62–1.24)
Lamichhane, 2019 [118]	Cohort study Women’s Health Initiative	76,853 women including 185 incident RA	Total coffee, for ≥4 cups/day versus none, HR 1.29 (0.84–1.98), P_trend_ = 0.16 Caffeinated coffee, for ≥4 cups/day versus none, HR 1.37 (0.84–2.23), P_trend_ = 0.37 Decaffeinated coffee, for ≥4 cups/day versus none, RR 1.76 (0.92–3.36), P_trend_ = 0.41 **Tea, for ≥4 cups/day versus none, RR 1.78 (0.83–3.82), P_trend_ = 0.03**
**Beverage**			
Pattison, 2004 [112]	Nested case-control EPIC Norfolk	88/176	Alcohol, >8.9 g/day versus none: OR 1.0 (0.6–2.0); *P*_trend_ = 0.9
Di Giuseppe, 2012 [119]	Cohort study (Swedish Mammography Cohort)	34,141 women including 197 incident RA	Regular alcohol drinkers versus occasional drinkers, RR 0.81 (0.59–1.11) Alcohol consumption, for ≥4 glasses/week versus <1 or never, RR 0.63 (0.42–0.96), ***P*_trend_ = 0.04**
Jin, 2013 [120]	Meta-analysis	195,095 participants including 1878 RA cases	High vs. no alcohol consumption, RR 0.99 (0.78–1.25) **Low-to-moderate vs. no alcohol consumption, 0.86 (0.78–0.94)**
Lu, 2014 [121]	Cohort study NHS I and II	193,206 women including 903 RA cases	Alcohol intake ≥10 g/day versus none: All RA patients, HR 0.74 (0.33–1.60), *P*_trend_ = 0.04; **seropositive RA patients, HR 0.81 (0.61–1.08), *P*_trend_ = 0.028;** seronegative RA patients, HR 0.63 (0.10–3.87), *P*_trend_ = 0.677
Hu, 2014 [122]	Cohort study NHS I and II	186,900 women including 857 RA cases	Sugar-sweetened soda consumption, ≥1 servings/d versus <1 serving/month: All RA, HR 1.33 (1.00–1.78), *P*_trend_ = 0.07; seropositive RA, HR 1.61 (1.15–2.30), *P*_trend_ < 0.01; seronegative RA, HR 0.90 (0.52–1.53), *P*_trend_ = 0.44
Sundström, 2015 [108]	Nested case-control	386/1886	Alcohol consumption (quartile 4 versus quartile 1): All RA, OR 0.99 (0.71–1.38); anti-CCP positive RA, OR 0.84 (0.57–1.25); RF-positive RA, OR 0.87 (0.61–1.25)
Hedström, 2019 [123]	Population-based case-control study (EIRA)	3353/2,836	Moderate consumption versus never: All RA, OR 0.49 (0.41–0.58); ACPA-positive RA, 0.43 (0.36–0.52); ACPA-negative RA, 0.58 (0.47–0.74) Three-way interaction between alcohol, smoking, and HLA-DRB1 SE with the risk of ACPA-positive RA
Nguyen, 2020 [111]	Cohort study E3N	62,629 women including 480 incident RA cases	Alcohol consumption, for tertile 3 versus tertile 1, HR 0.90 (0.68–1.20), *P*_trend_ = 0.41
**Dairy products**			
Sundström, 2019 [113]	Cohort study (Swedish Mammography Cohort)	35,600 women including 368 incident RA	Total dairy, for >6 servings/week versus ≤3, HR 1.09 (0.76–1.55) Milk, for >2 servings/week versus ≤0.5, HR 1.07 (0.80–1.43) Cheese, for >4 servings/week versus ≤1, HR 1.20 (0.81–1.76)
Nguyen, 2020 [111]	Cohort study E3N	62,629 women including 480 incident RA cases	Dairy product, for tertile 3 versus tertile 1, HR 1.12 (0.90–1.41), *P*_trend_ = 0.37
**Dietary pattern**			
Sundström, 2015 [108]	Nested case-control	386/1886	**Mediterranean diet score** (highest tertile versus lowest): All RA, OR 0.94 (0.68–1.29); anti-CCP-positive RA, OR 0.93 (0.64–1.36); RF-positive RA, OR 0.89 (0.63–1.27); smokers, OR 0.71 (0.40–1.25)
Hu, 2015 [109]	Cohort study NHS I and II	174,638 women including 913 incident RA cases	**Alternate Mediterranean diet score** (aMed), quartile 4 versus quartile 1: All RA, HR 0.98 (0.80–1.20), *P*_trend_ = 0.091; seropositive RA, HR 1.10 (0.85–1.42), *P*_trend_ = 0.51; seronegative RA, HR 0.80 (0.57–1.13), *P*_trend_ = 0.60
Hu, 2017 [124]	Cohort study NHS I and II	169,989 women including 1007 RA cases	**Dietary quality** measured by the 2010 Alternative Healthy Eating Index (AHAI-2010), quartile 4 vs. quartile 1 All population: All RA, HR 0.87 (0.73–1.05), *P*_trend_ = 0.15; seropositive RA, HR 0.92 (0.72–1.16), *P*_trend_ = 0.53; seronegative RA, HR 0.81 (0.60–1.09), *P*_trend_ = 0.14 Age <55 years: **All RA, HR 0.71 (0.54–0.94), *P*_trend_ = 0.009;** **seropositive RA, HR 0.65 (0.45–0.92), *P*_trend_ = 0.011;** seronegative RA, HR 0.83 (0.53–1.29), *P*_trend_ = 0.34 Age >55 years: No association
Johansson, 2018 [125]	Case-control EIRA	1721/3667	**Mediterranean diet score:** All population, OR 0.79 (0.65–-0.96); only smokers, OR 0.62 (0.40–0.95)
Sparks, 2019 [126]	Cohort study NHS I and II	173,560 women including 1185 incident RA cases	**Empirical inflammatory dietary pattern (EDIP),** quartile 4 (most inflammatory) vs. quartile 1 (least inflammatory) All population: All RA, HR 1.07 (0.89–1.28), *P*_trend_ = 0.54; seropositive RA, HR 1.01 (0.79–1.29), *P*_trend_ = 0.72; seronegative RA, HR 1.15 (0.86–1.53), *P*_trend_ = 0.59; Age <55 years: **All RA, HR 1.25 (0.94–1.65), *P*_trend_ = 0.09** Age >55 years: All RA, HR 0.93 (0.72–1.20), *P*_trend_ = 0.49
Nguyen, 2020 [111]	Cohort study E3N	62,629 women including 480 incident RA cases	**Mediterranean diet score**, per 1-unit: All RA, HR 0.96 (0.90–1.01); *P* = 0.11; never smokers, HR 1.00 (0.92–1.08), *P* = 0.79; - ever-smokers, HR 0.92 (0.85–0.99), *P =* 0.04

NHS, Nurses’ Health Study (USA); IWHS, Iowa Women’s Health Study; EIRA, Swedish Epidemiological Investigation of RA; RR, relative risk; OR, odds ratio; HR, hazard ratio; 95% CI, 95% confidence interval; RF, rheumatoid factor; ACPA, citrullinated peptide antibodies.
